# PROFILES OF DIGITAL LITERACY AMONG COMMUNITY-DWELLING KOREAN OLDER ADULTS: A LATENT PROFILE ANALYSIS

**DOI:** 10.1093/geroni/igad104.3536

**Published:** 2023-12-21

**Authors:** Jiyoung Shin, Hun Kang, Seongmi Choi, JiYeon Choi

**Affiliations:** Yonsei University, Seoul, Republic of Korea; Yale University School of Public Health, New Haven, Connecticut, United States; Yonsei University, Seoul, Republic of Korea; Yonsei University, Seoul, Republic of Korea

## Abstract

Digital divide exists among not only between older and younger generations but also within the older adult population. Understanding digital literacy profiles among older adults is important to identifying strategies for reducing digital divide among this population. This study aimed to identify profiles of digital literacy among community-dwelling older adults and examine associated factors. Data were collected from a national survey of 1016 community-dwelling older adults in Korea (average age 68.0±6.5 years, 47.8% male). Digital literacy was evaluated across three domains: ’Information and Communication’ (9 items), ’Contents Creation and Management’ (4 items), and ’Safety and Security’ (9 items). Latent profile analysis and multinomial logistic regression yielded three digital literacy profiles: “Low-level” (35.7%, n=363), “Middle-level” (39.2%, n=398), and “High-level” (25.1%, n=255). Compared to the low-level group, a higher likelihood of belonging to the middle level group was associated with younger age, higher education level, higher dependence on instrumental activities of daily living, longer digital device use, and participating in offline social activities. Similarly, a higher likelihood of belonging to the high-level group was associated with younger age, higher education level, higher dependence on instrumental activities of daily living, longer digital device use, better physical well-being, doing daily physical exercise, and greater social support. These findings suggest characteristics associated with lower digital literacy, including older age, lower education level, and reduced functional independence. To promote digital literacy in older adults, potential strategies encompass tailored access and guidance for digital device use specifically tailored to older adults, alongside efforts to nurture social connectedness.

